# Wild plants and fungi sold in the markets of Yerevan (Armenia)

**DOI:** 10.1186/s13002-020-00375-3

**Published:** 2020-05-19

**Authors:** Siranush Nanagulyan, Narine Zakaryan, Nune Kartashyan, Renata Piwowarczyk, Łukasz Łuczaj

**Affiliations:** 1 Department of Botany and Mycology, Yerevan State University, A. Manoogian, 1, 0025 Yerevan, Armenia; 2 Department of Microbiology and Parasitology, Institute of Biology, Jan Kochanowski University, Uniwersytecka 7, 25-406 Kielce, Poland; 3 Institute of Biology and Biotechnology, University of Rzeszów, Pigonia 1, 35-310 Rzeszów, Poland

**Keywords:** Ethnobotany, Ethnomycology, Open-air markets, Caucasus, Edible plants and fungi, Food plants, Medicinal plants

## Abstract

**Background:**

The aim of the study was to record wild plants and fungi sold in the capital of Armenia. This is the first large market survey in the Caucasus region. The area of the Caucasus is characterised by a very high diversity of climates, flora and languages which results in very rich traditions of plant use.

**Methods:**

Interviews were conducted and photos and voucher specimens were taken during multiple visits made over 4 years. We studied 37 locations and 136 people were interviewed.

**Results:**

As many as 163 plant species, belonging to 44 families and 110 genera, were recorded on Yerevan markets. This included 148 wild food species, 136 medicinal species, 45 species sold for decoration, 15 species of wood and 9 species of insect repellents. Also 14 wild species of fungi were sold, including 12 food species.

**Conclusions:**

The list of plants sold in the markets of Yerevan is very extensive and diverse, and includes many species of wild fruits, vegetables and medicinal plants, some of them never listed in ethnobotanical directories before. A characteristic feature of this market is a large representation of lacto-fermented products. Some of the species sold in Yerevan have never been reported as human food either in wild edible plant word lists or in ethnobotanical publications, e.g. *Angelica tatianae*, *Ferulago setifolia* and *Heracleum chorodanum*. Fungi are also well represented.

## Background

The Caucasus is one of the richest regions of Eurasia in terms of biocultural diversity as well as being one of the globe’s most important biodiversity hotspots [1]. In the Caucasus, a large number of climate types and high altitudinal variation is combined with high ethnic diversity. The Caucasus Mountains host more languages than the rest of Europe [2, 3]. The large diversity of economic plants and their uses was recorded by botanists and agriculturalists from the Soviet Union, including Grossgeim and Vavilov [4, 5]. Presently a new era of detailed ethnobotanical exploration of the Caucasus has begun. It consists of detailed ethnobiological exploration (e.g. [6–18]). In-depth local studies have revealed many interesting, and sometimes unique, plant uses.

Open-air markets hold an important position for ethnobotanists and ethnomycologists. Ethnobotanical studies of open-air markets are a frequent topic of ethnobotanical enquiry, as they are places where one can usually find plants that are the most important to a given culture, e.g. commonly eaten fruits, vegetables or medicinal plants (e.g. [18–56]). The oldest known ethnobiological market surveys were carried out in the 1920s by Pénzes in Hungary [24, 25] and Polish researchers: in the 1920s in Wilno/Vilnius (now the capital of Lithuania) by Muszyński [21] and in the 1930s in Poznań, Poland (Szulczewski) [22, 23]. Another important early work based on market surveys is the study of Bye from Mexico [19].

The ethnobiological diversity of organisms sold in open-air markets in the Caucasus has only been explored in two papers from Georgia, one about medicinal plant mixes in Borjomi [18], the other on wild vegetables sold in the markets of Kutaisi [10].

There has always been a great demand for wild plants amongst the Armenian population. They have benefited from the use of various wild plants since ancient times, and they have passed on their traditions from generation to generation. The herbs of the Armenian Highlands were highly praised by the Greek physician, pharmacologist, botanist and author of *De Materia Medica*, Pedanius Dioscorides [57]. Traditionally, Armenians have used plants as food, medicine, fuel, construction material, dyes for carpet yarns, insect repellent and for other purposes.

The Armenian flora is represented by around 3800 species of vascular plants from 160 families and 913 genera, including 146 endemic species. It is estimated that about 20% of the species composition of the flora of Armenia is in use by its population [16]. Amongst these plants, about 380 species have medicinal applications used in traditional folk medicines, approximately 90 species are used in scientific medicine, and around 320 species are traditionally used edible plants. It is estimated that out of the 1400 species of macroscopic fungi in the country, at least 300 edible, 60 poisonous and more than 120 species with medicinal properties have been recorded. However, the traditional use of mushrooms in Armenia is little studied [58].

## Methods

### Aim of study

The aim of the study was to document the taxonomic diversity and uses of the wild plants and fungi sold in the capital of Armenia, Yerevan.

### Study area

Armenia is a southern Caucasian republic with a total area of 29,740 km^2^, bordered by Georgia, Azerbaijan, Turkey and Iran. Armenia is a mountainous country, dominated by a series of mountain massifs and valleys, with its lowest point at 375 m above sea level and culminating at 4095 m (Mt Aragats—extinct volcano) with an average elevation of 1850 m [59]. About 90% of the country lies at an altitude of over 1000 m above sea level and is located in a seismically active area. It is home to Sevan, the largest lake in the Caucasus (area 1240 km^2^), a tectonic ditch at an altitude of 1900 m above sea level. The diversity of landscapes, climates (6 basic types, from dry subtropical up to extreme alpine) and orography is an important determinant of Armenia’s vegetation. The lower mountain belt (375–1200 m) is represented by semi-desert or phryganoid formations (i.e. vegetation dominated by small, fragrant, prickly semishrubs of the Lamiaceae, Asteraceae family and *Astragalus*, *Euphorbia* genera), gypsophilous or halophilous vegetation, salt marsh areas, as well as the Transcaucasian sand desert. The middle and upper mountain belts (1200–2200 m) are characterised by diversified steppe and forest vegetation, meadow-steppes, shrub steppes and thorny cushion (tragacanth) vegetation. The altitudinal span of the forest belt varies from 500 to 1500–2000 m. The subalpine and alpine belts (2200–4000 m) are covered by tall-grass vegetation, meadows and carpets, with an abundance of biocoenoses, rich species composition and a high level of endemism [60–63].

Yerevan, the capital of Armenia, dates back to the 8th century BC and is one of the world’s oldest continuously inhabited cities. It is situated along the Hrazdan River and is the administrative, cultural, and industrial centre of the country, where more than half of the country’s inhabitants are concentrated. According to an official estimate from 2016, the city has a current population of 1,073,700 [64]. The city used to be an important centre for trade and came under siege from the Romans, Arabs, Mongols, Turks, Persians, Georgians, and Russians. These various foreign influences, mixed and evolving for centuries, are still visible today, e.g. in the architecture, traditions, and of course in the use of wild plants or spices in cooking.

The city of Yerevan is divided into 12 administrative districts, and each of them has its own market. The largest markets are located in the Kentron, Arabkir and Malatia-Sebastia districts. Yerevan’s surroundings belong to the Yerevan Floristic Region, with vertical altitudes from 700 to 1700 m above sea level. The main floristic inventory work focused on the region around Yerevan was performed between the 1950s and 1980s. During a period of economic blockade and energy crisis (1992-1995), woody vegetation was extensively cut down, especially in the vicinity of hills around Yerevan, which has led to the increased erosion of soils on hillsides.

The flora of the Yerevan Floristic Region counts 1920 species, from which 46 species are endemic, and 144 species included in the Red Book of Armenia [16, 65]. The low mountain belt of the region (700–1200 m) is covered by semi-desert or phryganoid formations, gypsophilous and halophilous vegetation. There are salt marsh areas as well as the Transcaucasian sand desert. The middle and upper mountain belts (1200–1700 m) are characterised by various kinds of steppe vegetation, shrub steppes and thorny cushion (tragacanth) vegetation [16, 66].

### Data collection

**Fig. 1:**
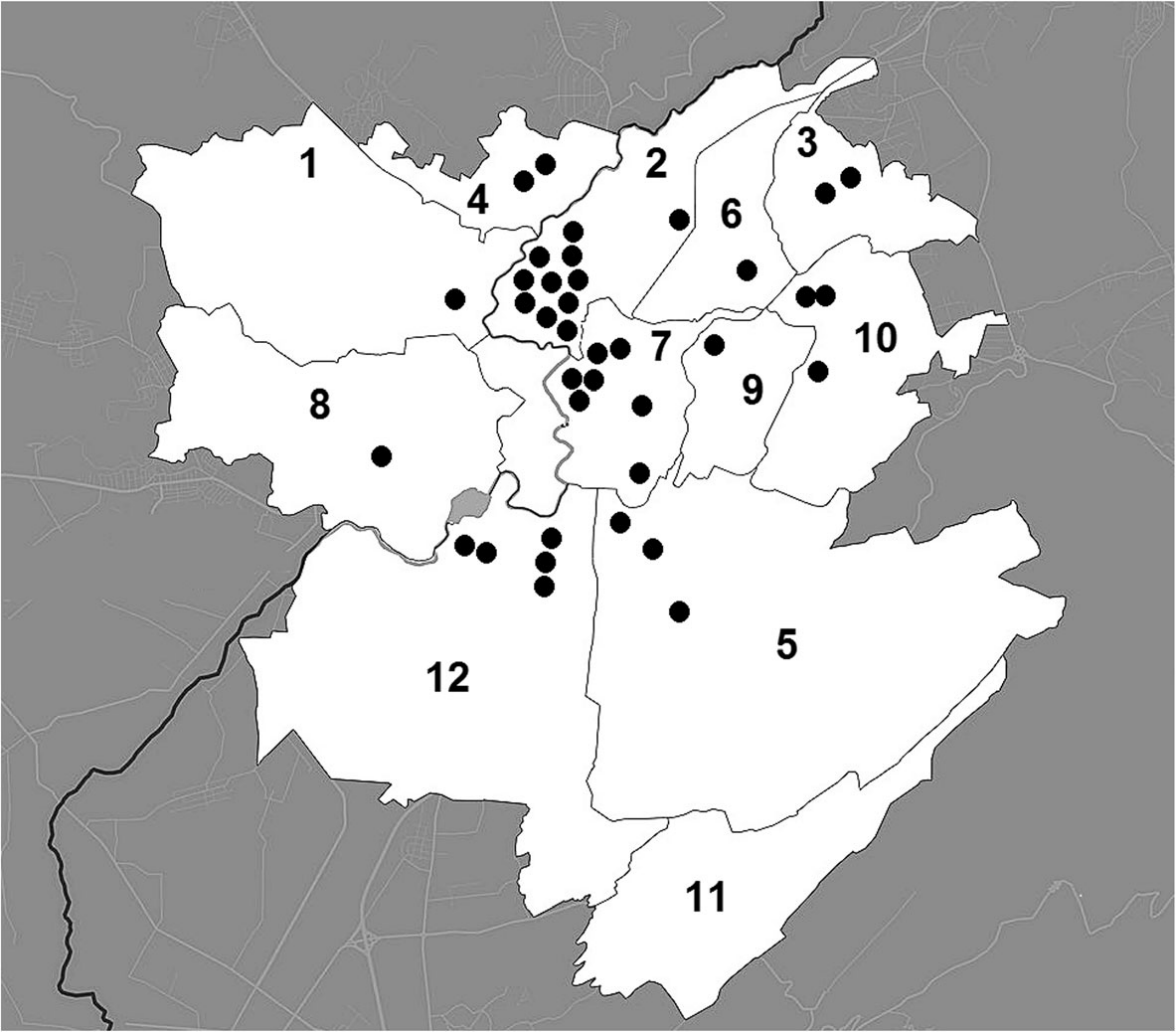
Distribution of studied market places (black dots) in administrative districts of Yerevan: 1. Ajapnyak, 2. Arabkir, 3. Avan, 4. Davtashen, 5. Erebuni, 6. Kanaker-Zeytun, 7. Kentron, 8. Malatia-Sebastia, 9. Nork-Marash, 10. Nor Nork, 11. Nubarashen, 12. Shengavit

Ethnobotanical and ethnomycological information was gathered using unstructured or semi-structured interviews and focus group discussions with city population and sellers in the markets. The observations were made in Yerevan between 2016 and 2019 in 37 open-air and farm markets, supermarkets, streets shops and other locations where wild plants and fungi were sold (Appendix 1; Fig. 1). The interviews were conducted in every month throughout the year. During the interviews, fresh or dried plant and fungi samples were collected as voucher specimens where possible. In some cases, the plants were also collected from nature. A total of 136 respondents were interviewed. The age of them varied from 20 to 80. Most respondents were women (83%) and only 17% were men. Respondents were asked about the traditional uses of the plants and fungi that were for sale, local names of species, their therapeutic effects and methods of preparation and cooking.

The plants and fungi were identified by the authors using the Flora of Armenia [67], the Mycoflora of Armenia Soviet Socialist Republic [68] and Cap Fungi of Armenia [69]. Voucher specimens were deposited at the Herbarium of the Yerevan State University (ERCB—plants, ERHM—fungi). Plant names were updated according to the Plant List [70]. Fungi names follow Index Fungorum [71].

Some of the taxa included in the list of species (Appendix 2) are often cultivated (e.g. *Morus, Ficus, Punica*). However, we included them in the list because they also often occur in a wild or semi-wild state.

## Results

**Fig. 2:**
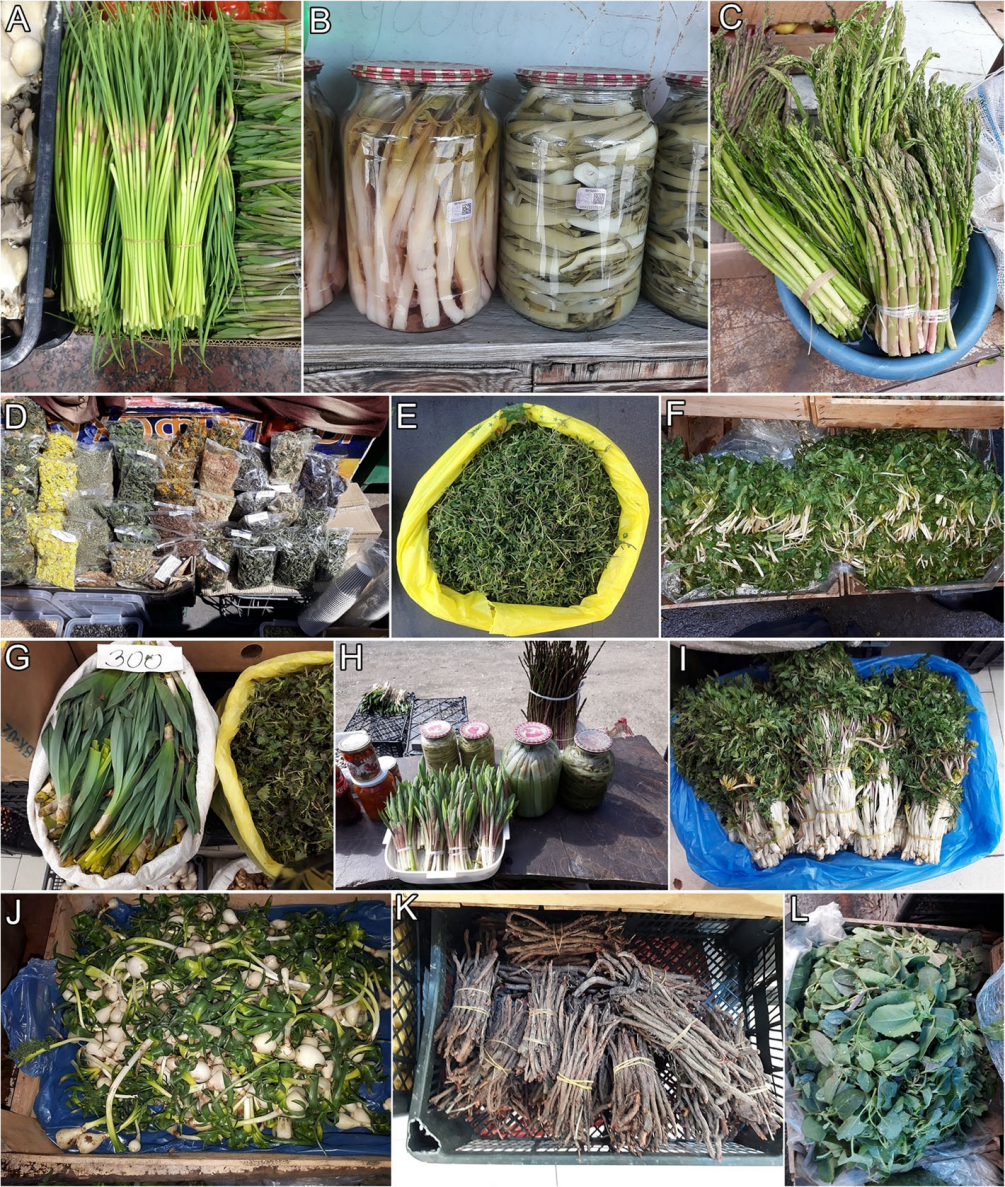
Wild plants sold in the markets of Yerevan **a***Allium victorialis*. **b***Chaerophyllum bulbosum* (pickled) and *Bilacunaria microcarpa* (pickled). **c***Asparagus officinalis*. **d***Helichrysum* sp., *Thymus* sp., *Pinus kochiana*, *Hypericum* sp., *Tanacetum* sp., *Salvia* sp., *Valeriana officinalis*, *Cichorium intybus*, *Inula helenium*, *Mentha piperita*, *Leucanthemum vulgare*. **e***Ziziphora clinopodioides*. **f***Falcaria vulgaris*. **g***Eremurus spectabilis*, *Urtica dioica*. **h***Polygonatum orientale*. **i***Chaerophyllum aureum*. **j***Ornithogalum montanum*, **k***Rubia tinctorum* roots. **l***Chenopodium album*

**Fig. 3:**
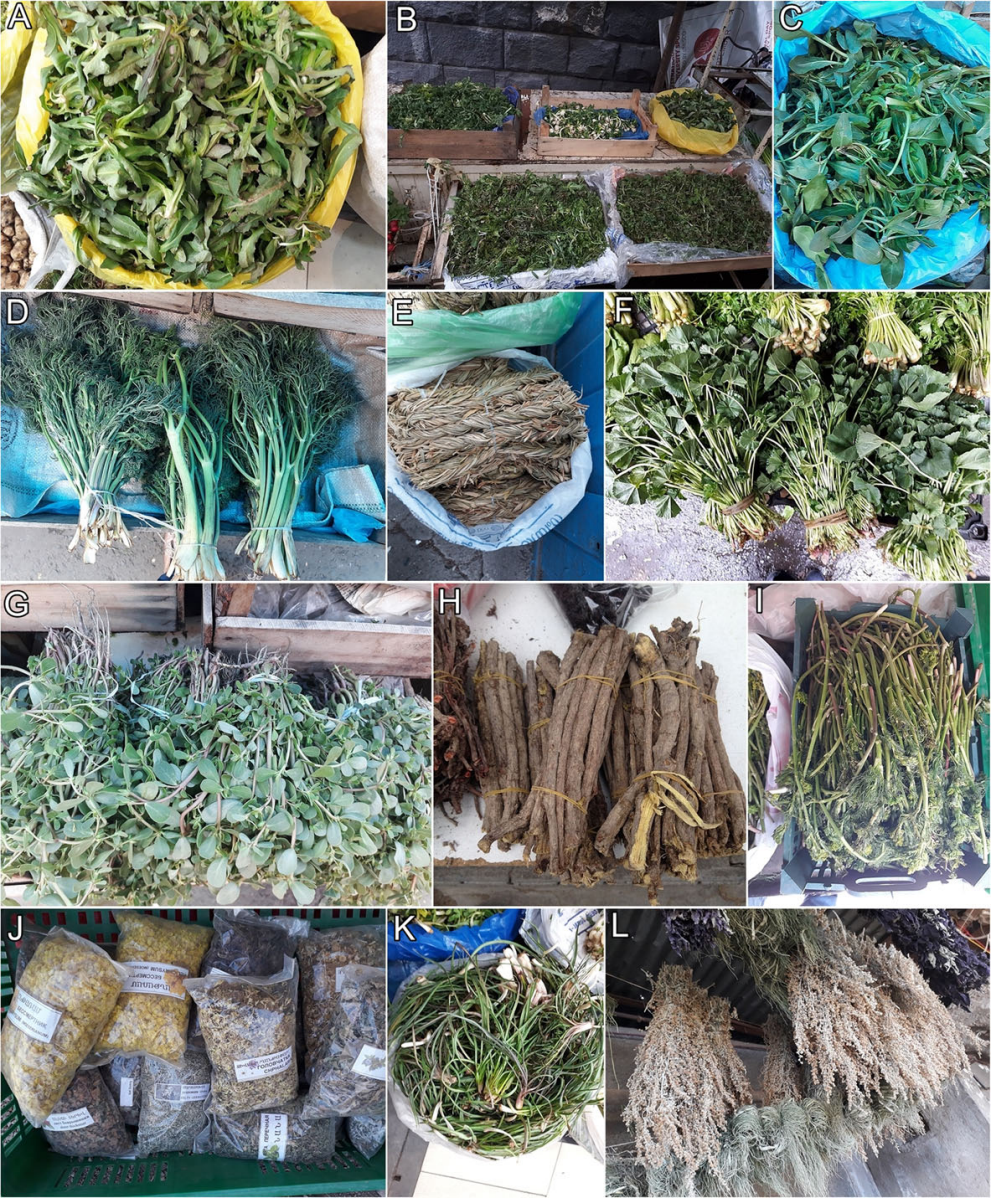
Wild plants sold in the markets of Yerevan. **a***Lactuca serriola*. **b***Urtica dioica*, *Ornithogalum montanum*, *Senecio vernalis*. **c***Lepidium draba*. **d***Bilacunaria microcarpa*. **e***Ornithogalum hajastanum* dried. **f***Malva neglecta*. **g***Portulaca oleracea*. **h***Glycyrrhiza glabra*. **i***Ferulago setifolia*. **j***Teucrium polium*, *Cephalaria gigantea*, *Crataegus* sp., *Helichrysum rubicundum*. **k***Tragopogon* sp., **l***Artemisia absinthium*, *Equisetum arvense*

**Fig. 4:**
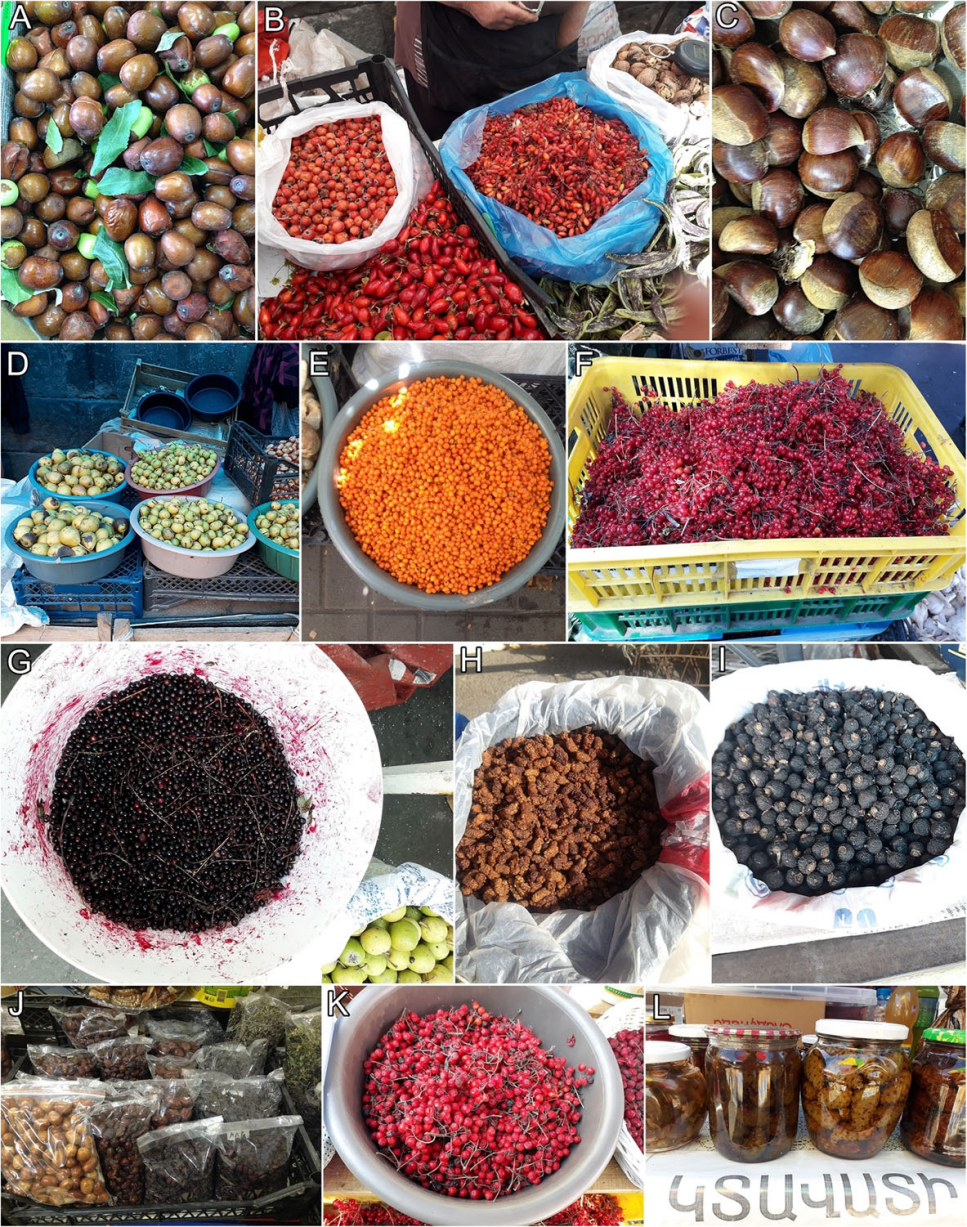
Wild fruits and nuts sold in the markets of Yerevan. **a***Ziziphus jujuba*. **b***Berberis vulgaris*, *Rosa canina*, *Crataegus orientalis*. **c***Castanea sativa*. **d***Pyrus calicifolia*, *P. caucasica*. **e***Elaeagnus rhamnoides*. **f***Viburnum opulus*. **g***Ribes petraeum*. **h***Morus alba*. **i***Rosa spinosissima*. **j***Elaeagnus angustifolia*, *Rosa* sp., *Cornus mas*. **k***Sorbus aucuparia*. **l***Pinus kochiana* jam and tincture of female cones

**Fig. 5:**
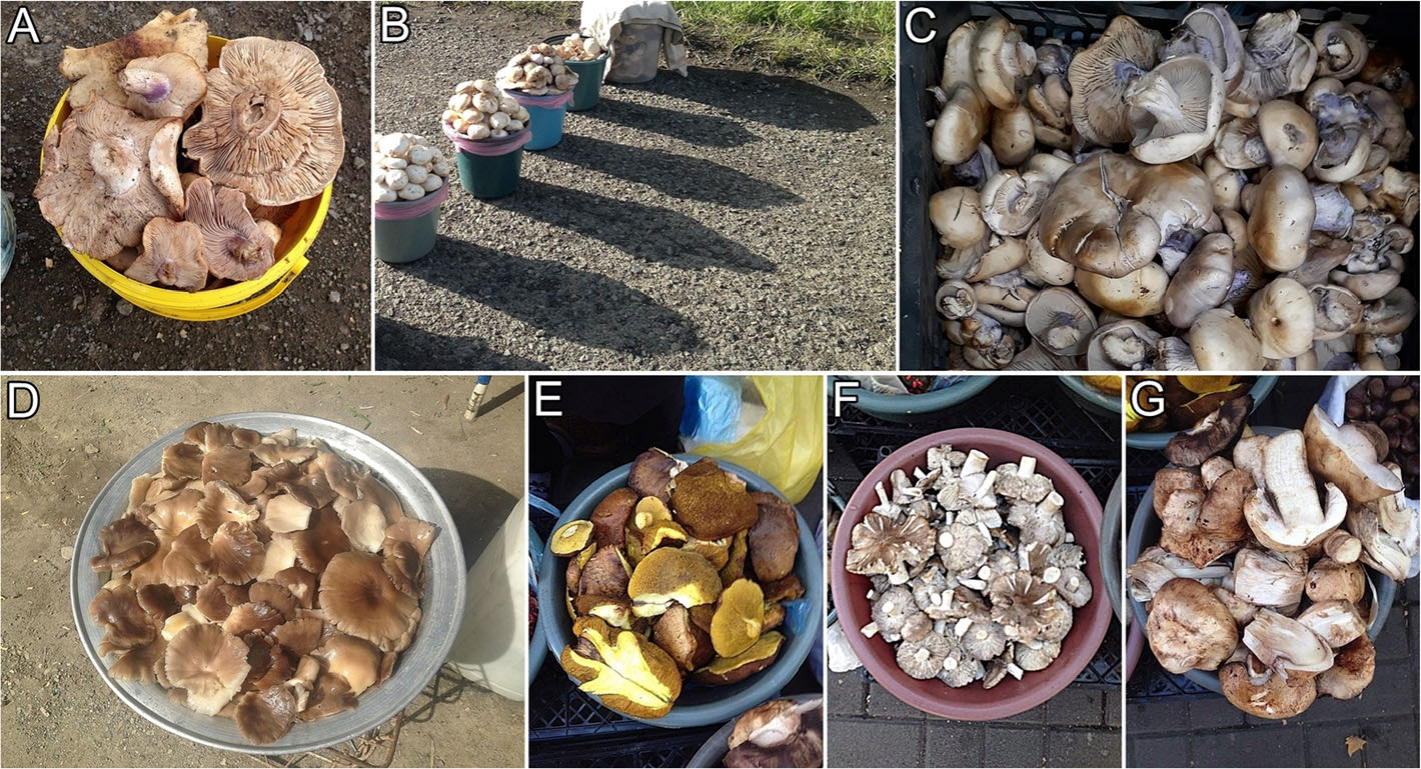
Wild mushrooms sold in the markets of Yerevan. **a***Lepista personata*. **b***Agaricus campestris* and *Lepista personata*. **c***Lepista nuda*. **d***Pleurotus ostreatus*. **e***Suillus granulatus*. **f***Tricholoma terreum*. **g***Agaricus bisporus*

Altogether 163 plant species have been recorded on Yerevan markets during this study (Appendix 2; Figs. 2, 3 and 4). They belong to 44 families and 110 genera. The most common plant families are Asteraceae (20%), Rosaceae (14%), and Apiaceae (11%). *Tragopogon* and *Crataegus* (both 6 species) are used the most. As many as 17 species of fungi are sold in open-air markets including 14 species collected from the wild and three species cultivated for food. Most of the mushrooms, namely 12 species, are wild species sold for culinary purposes (Appendix 2; Fig. 5).

As many as 148 plant species are sold for food, 136 species are sold as medicine or are food species with perceived medicinal values, 45 species are decorative plants, 15 plants are a source of wood and nine species are used as an insect repellent.

The largest category of species sold in the markets is those used for food. The most commonly sold and used food species are *Rumex crispus*, *Chaerophyllum bulbosum*, *Astrodaucus orientalis*, *Malva neglecta*, *Falcaria vulgaris*, *Asparagus officinalis*, *A. verticilata*, *Eremurus spectabilis*, *Urtica dioica* and *Polygonatum orientale* (for authority names cited in the text, see Appendix 2 for plants and Table 1 for fungi).

**Table 1:** Fungi sold in Yerevan’s markets

Species	Voucher number (ERHM)	Widely used Armenian name	Ways of use
*Agaricus arvensis* Schaeff.	**10764**	Shampinion	CUL: Fried, boiled
*Agaricus bisporus* (J.E. Lange) Imbach^a^	**10683**	Shampinion	CUL: Fried, boiled, lacto-fermented
*Agaricus campestris* L.^a^	**10629**	Shampinion	CUL: Fried, boiled, lacto-fermented
*Armillaria* sp.	**10190**	Kotchghasunk	CUL: Fried, boiled
*Calocybe gambosa* (Fr.) Donk	**11080**	Sharqasunk, garan dmak	CUL: Fried, boiled
*Cantharellus cibarius* Fr.	**10774**	Aghvesasunk	CUL: Fried, boiled, lacto-fermented
*Fomes fomentarius* (L.) Fr.	**11079**	Abetasunk	DEC: Fruiting bodies used as decorative elements
*Ganoderma lucidum* (Curtis) P. Karst.	**10424**	Laqapat abetasunk	MED: Sold to be used in Chinese medicine. DEC: Fruiting bodies used as decorative elements
*Lactarius deliciosus* (L.) Gray	**11081**	Sheklik	CUL: Fried, boiled, lacto-fermented
*Lactarius deterrimus* Gröger	**10328**	Sheklik	CUL: Fried, boiled, lacto-fermented
*Lepista nuda* (Bull.) Cooke	**10692**	Kapuyt sunk	CUL: Fried, boiled
*Lepista personata* (Fr.) Cooke	**10694**	Kapuyt votikov sunk	CUL: Fried, boiled
*Marasmius oreades* (Bolton) Fr.	**10633**	Kochghasunk dashti	CUL: Fried, boiled
*Pleurotus eryngii* (DC.) Quél.	**10783**	Tagavorakan akandjasunk	CUL: Fried, boiled, lacto-fermented
*Pleurotus ostreatus* (Jacq.) P. Kumm.^a^	**10782**	Akandjasunk, kakhasunk, tsari sunk, vostresunk	CUL: Fried, boiled, lacto-fermented
*Suillus granulatus* (L.) Roussel	**10502**	Yuxhasunk	CUL: Fried, lacto-fermented
*Tricholoma terreum* (Schaeff.) P. Kumm.	**10604**	Sharqasunk mokhraguyn	CUL: Fried, lacto-fermented

^a^Artificially cultivated

**Fig. 6:**
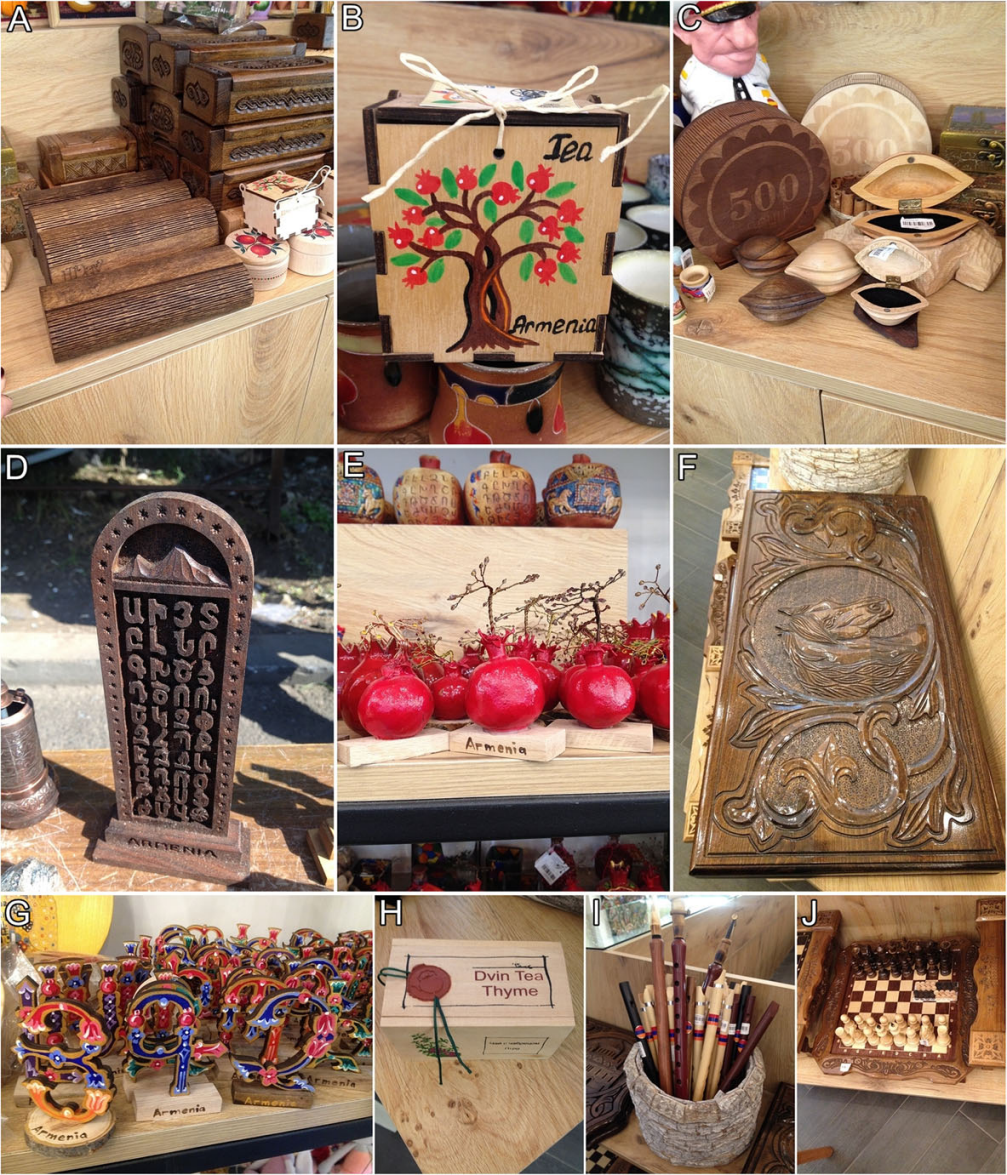
Handicrafts made from wild woods sold in the markets of Yerevan. **a***Prunus armeniaca* case for glasses. **b***Fagus orientalis* wooden box for tea. **c** Handicrafts of *Prunus armeniaca*—moneyboxes and jewellery boxes. **d***Prunus armeniaca*, wooden handicraft. **e** Pomegranates from wood. **f***Fagus orientalis* wood backgammon. **g** Handicrafts of *Prunus armeniaca*. **h***Prunus divaricata* wooden box for tea. **i** Wooden musical instruments (duduk, shvi). **j***Fagus orientalis* and *Prunus divaricata* wooden chess

Wild food plants are used for a variety of dishes (Fig. 6). Young leaves of *Stellaria media*, *Anthriscus nemorosa*, *Capsella bursa-pastoris*, *Urtica dioica*, *Mentha longifolia*, *Allium* spp., *Tragopogon* spp., and *Rumex* spp. serve as filling for pies called *zhingyalov hats*, a type of flatbread stuffed with finely diced herbs. Young leaves of *Vitis vinifera* are used to wrap dolma (stuffed leaves with meat). Young leaves of *Chaerophyllum aureum*, fried with eggs, are called *tapakats shushan* and a similar dish made with *C. bulbosum* called *tapakats mandak*. Young leaves of *Falcaria vulgaris* are also commonly fried with eggs for a dish called *tapakats sibekh*. Fruiting bodies of *Lepista personata* and *Agaricus campestris* are combined with *Triticum dicoccon* (emmer wheat) for the Armenian pilav*—acharov plav*.

Inhabitants of the city also use some plants for salads, e.g. *Urtica dioica*, *Portulaca oleracea* and *Rumex acetosa*. Soups are made with different species of *Malva* and *Rumex*, and with *Puschkinia scilloides*. A larger variety of dishes is prepared from *Asparagus officinalis*, *A. verticillatus*, *Astrodaucus orientalis*, *Capsella bursa-pastoris*, *Chaerophyllum aureum*, *C. bulbosum*, *Eremurus spectabilis*, *Falcaria vulgaris*, *Hippomarathrum microcarpum*, *Lactuca serriola*, *Lepidium draba*, *L. latifolium*, *Ornithogalum hajastanum*, *Polygonatum giaberrimum*, *P. multiflorum*, *P. orientale*, different species of *Tragopogon* and *Rumex.*

**Fig. 7:**
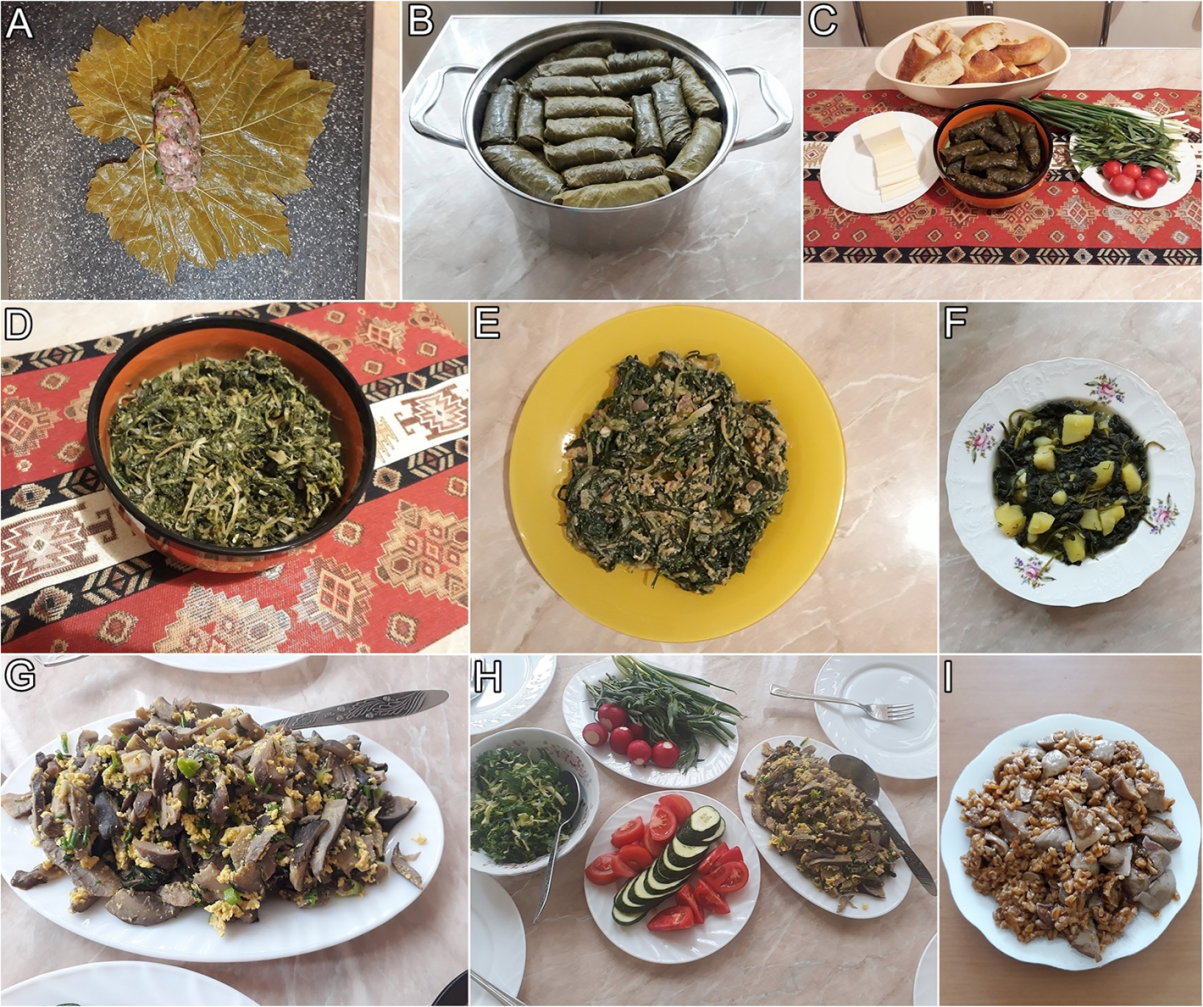
Selected dishes using wild plants and mushrooms from the markets of Yerevan. **a**, **b**, **c** Young stuffed leaves of grape *Vitis vinifera* with meat for dolma. **d***Falcaria vulgaris* fried. **e***F. vulgaris* fried with eggs. **f***Malva neglecta* (soup with potatoes)—Pipertov apur. **g***Pleurotus ostreatus* with eggs. **h** fried *Ornithogalum montanum* (left) and fried *Pleurotus ostreatus* with eggs (right). **i***Lepista personata* with *Triticum dicoccon* (emmer)—Acharov plav

*Artemisia absinthium, Berberis vulgaris, B. orientalis, Carum carvi, Origanum vulgare, Thymus* spp. and *Ziziphora rigida* are used as flavouring. Different species of *Thymus* and *Allium* are commonly used for flavouring cheese and curd.

Numerous species are used to make recreational teas, e.g. *Rosa* spp.*, Mentha longifolia, Cephalaria gigantea, Origanum vulgare* and different species of *Thymus*. *Juglans regia*, *Prunus armeniaca*, *Corylus avellana* and seeds of *Cannabis sativa* are used as edible nuts. As for berries and fruits, locals buy *Cornus mas*, *Elaeagnus angustifolia*, *E. orientalis*, *E. rhamnoides*, *Ficus carica*, *Morus alba*, *M. nigra*, *Prunus armeniaca*, *P. divaricata*, *Punica granatum*, *Ribes alpinum*, *Viburnum opulus*, *Ziziphus jujuba and* different species of *Crataegus*.

The species which are sold and used most frequently as medicinal remedies in the city of Yerevan include *Artemisia absinthium*, *Hypericum perforatum*, *Mentha longifolia*, *Origanum vulgare*, *Teucrium polium* and three species of genus *Thymus*—*T. kotschyanus, T. rariflorus, T. transcaucasicus*. The most common types of remedies are those for the treatment of digestive disorders, the common cold and other respiratory problems.

An important segment of wild plants is the wood (Fig. 7) used for manufacturing musical instruments, like *Prunus armeniaca* (used to make *duduk*, *tar*, *qyamancha*, and *zurna*)*, P. divaricata* (for *saz*) and different national handicrafts and souvenirs (the wood of *Fagus orientalis*, *Juglans regia* and *Prunus armeniaca)*. Fruit bodies of *Fomes fomentarius* and *Ganoderma lucidum* commonly are used as decorative elements.

## Discussion

The presented list of useful plants sold in Yerevan consists of diverse categories, including both food and medicine, as well as other smaller categories. This diversity of plant uses brings studies of both southwest and southeast Asian markets to mind. In Table 2, we put together other publications on the ethnobotany and ethnomycology of markets in different parts of Eurasia. Out of studies concerning more than one plant category, the largest number of species was recorded in the market of Bodrum, Turkey, with as many as 390 species [29]. In Turkey, similarly to Yerevan, large numbers of wild vegetables and medicinal plants are sold. The number of edible plants recorded was 143 but the number of fungi species was 7 (compared to 17 in our study). Unfortunately, we do not have lists of plants from other large towns of the Caucasus region to make local comparisons. In Kutaisi in Georgia, Łuczaj et al. [10] have recorded sales of 26 species of wild vegetables, while the number of species sold in Yerevan is much larger, with as many as 65 different species. In contrast to Yerevan, few wild vegetables are sold in the open markets of Central Europe, e.g. Poland and Hungary [41, 53] (mainly *Rumex* and *Allium ursinum*), and only a small portion of medicinal plants is sold [41, 53], though in the early 20th century, the medicinal sector in the markets of Poland was an important part of open-air markets [21–23]. But still, even in the 1920s and 1930s, the number of edible and medicinal plants for sale was lower than in contemporary Yerevan. On the other hand, the number of fungi sold in the markets of central Europe is higher than in Yerevan. For example, in southeastern Poland Kasper-Pakosz et al. [53] recorded the sales of 32 species, including 20 wild ones. Earlier in the 1930s, Szulczewski [22] recorded as many as 56 fungi species in Poznań. Of course, the number of species of fungi sold in Yerevan is still quite high—higher than in most south Asian markets. The large choice of wild vegetables and wild edible fungi must reflect the strongly herbophilic (sensu Łuczaj [72]) and mycophilic [73] approach of the inhabitants of Yerevan.

**Table 2:** Ethnobotanical inventories carried out in markets in Eurasia listed chronologically

Study	Country, region, city	Number of markets	Year	Surveyed categories or parts of plants	Number of species reported
Pénzes [24, 25, 41]	Hungary, Pest (now Budapest)	Not specified	1922-1925	Wild plants	89
Muszyński [21]	Poland (now Lithuania),Vilnius	1	1927	Medicinal plants	113 plants, 4 fungi
Szulczewski [22]	Poland, Poznań	Not specified,	Before 1933	Edible fungi	56
Szulczewski [23]	Poland, Poznań	Not specified	1933	Medicinal plants	79
Pemberton et al. [33]	South Korea, Seul	3	1989-1995	Wild and cultivated vegetables and fruits	112
Hamayan et al. [39]	Pakistan, cities of Kalam, Madyan, Mingora, Peshawar, Rawalpindi and Lahore	6	2002	Medicinal	44
Xu et al. [34]	China, Yunnan, Xishuanbanna	14	1996-2001	Medicinal and edible	284 plants, 18 fungi
Ertug [29]	Turkey, Mugla, Bodrum	1	1999-2002	All useful plants, including medicinal, ritual, edible	390, including 143 edible and 7 fungi
Hanlidou et al. [27]	Greece, Thessaloniki	1 (18 stalls)	2002	Medicinal plants	172
Kar and Borthakur [47]	India, Assam, Karbi Anglong	Not specified	2003	Wild vegetables	29
Karousou et al. [26]	Cyprus	15 shops, 3 markets, 3 cities	2005-2008	Medicinal plants	57
Mati and de Boer [37]	Iraq, the Kurdistan Autonomous Region, Erbil, The Qaysari bazaar	21 herbalist shops	2008-2010	Medicinal plants	83
Salam et al. [48]	India, Ukhrul District of Manipur	3	2009-2010	Leafy vegetables	55
Shirai et al. [35]	Thailand, Khon Kaen (Bang Lam Phu)	1 large and 10 small, 139 stalls	2006	Wild edibles	54 plants, 6 fungi
Dogan et al. [30]	Turkey, Izmir	18	2009-2011	Wild edible plants	46
Amiri et al. [40]	Iran, Mashhad	Over 600 shops	2011-2012	Medicinal plants	269
Łuczaj et al. [28]	Croatia, Dalmatia, all major 11 coastal cities and towns	11	2012	Wild leafy vegetables	37
Dogan and Nedelcheva [32]	SE Bulgaria (4 towns) and NW Turkey (3 towns)	7	2011-2013	Medicinal and wild edible plants	41 in total, 34 medicinal, 15 edible
Dogan and Nedelcheva [32]	SE Bulgaria (4 towns) and NW Turkey (3 towns)	7	2011-2013	Medicinal and wild edible plants	41 in total, 34 medicinal, 15 edible
Silalahi i in [43].	Indonesia, Kabanjahe (Sumatra)	1	2015	Medicinal plants	344
Vlkova et al. [38]	Kyrgyzstan	2	2012	Plants, mainly edibles	20
Konsam et al. [36]	India, Manipur	20	2012-2014	Wild vegetables	68
Łuczaj et al. [10]	Georgia, Kutaisi	2	2014-2015	Leafy vegetables	26
Sucholas [44]	Poland, Poznań	1	2013	Medicinal and culinary herbs	21 typically medicinal herbs and 23 culinary potted herbs
Kasper-Pakosz et al. [53]	Poland, Podkarpackie, 4 cities	4	2013-2015	All plant categories, edible fungi	468 species of plants were recorded, only 30 species from the wild, 32 species of edible fungi (including 30 wild ones), most species are cultivated ornamentals
Li et al. [42]	Chiny, Guangdong, Chaosahn	12 markets, 83 stalls	2013-2015	Herbal teas	186
Bussmann et al. [18]	Georgia, Borjomi	1	2013-2015	Medicinal plant mixes	40
Dénes [41]	Hungary, Pecs	A few	2012-15	All wild plants	130 in total, including 98 ornamentals, 10 species of wild vegetables, 6 species offlowers, 18 species offruits, 38 fungi
Zhang et al. [49]	China, NW Yunnan, Dali	1	In 1987/88 and 2012/13	Medicinal plants	505 versus 709 species
Sulaini and Sabran [55]	Malaysia, Johor, Baty Pahat	Not specified	Not specified	Medicinal plants	120
Nguyen et al. [46]	Vietnam, Son La	32	2016-2018	Medicinal plants	99
Franco et al. [57]	Tamu Kianggeh of Bandar Seri Begawan, Brunei Darussalam	1	2019	Food plants	104 species of fruits and vegetables (mainly cultivated)
This study	Armenia, Yerevan	37	2016-2019	Various plant and fungi categories	163 plant species, including 148 wild food species, 136 medicinal species, 45 species sold for decoration, 15 species of wood and 9 species of insect repellents;. 17 species of fungi including 15 food

**Fig. 8:**
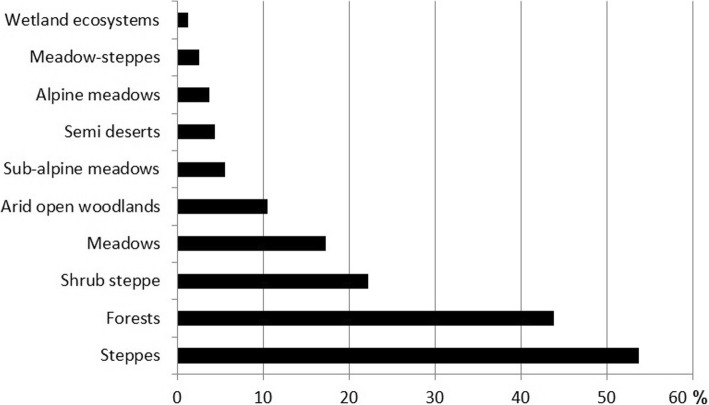
Percentage of plants coming from different habitat zones

Most of the plants sold in the markets are relatively common. The main source of plants are the surrounding steppes and forests (Fig. 8). Only few species come from high altitudes or (semi)deserts. However, four Armenian Red List species have been recorded on Yerevan markets [65]. This includes three plant species: *Acorus calamus* with endangered status—EN B 1 ab (i, ii, iii, iv) + 2 ab (ii, iii); *Castanea sativa*, endangered—EN B 1 ab (iii) + 2 ab (iii) and *Ferula szowitsiana*, vulnerable—VU B 1ab (ii, iii, iv) + 2 ab (ii, iii, iv), as well as one species of fungus, *Pleurotus eryngii,* vulnerable*—*VU. We suspect that *F. szowitziana, A. calamus* and *P. eryngii* can be affected by harvesting from the wild, as *C. sativa* is cultivated.

A characteristic feature of Yerevan markets is the many species of lacto-fermented products sold in jars. These include many wild plant species. In our study, we recorded 26 species of plants preserved in this way, including as many as 11 species from the Apiaceae. The wide use of wild Apiaceae as food, e.g. from the genera *Heracleum, Anthriscus,* and *Chaerophyllum*, seems to be a characteristic feature of the whole Caucasus area (e.g. [6, 14, 17, 74] and Anna Janicka-Galant, Łódź, pers. comm.). We recorded also 9 species of fungi, which are used as lacto-fermented products, e.g. from the genera *Agaricus, Lactarius* and *Pleurotus*. The context of fermented foods and their documentation is important due to their growing popularity and possible health benefits [75, 76].

Apart from wild foods that are commonly found in Caucasian, European and south Asian markets, some of the species sold in Yerevan have never been reported as human food either in wild edible plant word lists or in ethnobotanical publications. These include some plants from the Apiaceae family: *Angelica tatianae*, *Ferulago setifolia* and *Heracleum chorodanum*. Two species (*Heracleum antasiaticum* and *Bilacunaria microcarpa*), also from Armenia, have only recently been reported as food a few weeks ago [17].

Surprisingly, *Senecio leucanthemifolius* subsp. *vernalis* is sold as a wild vegetable. This genus of ragworts is famous for a high content of pyrrolizidine alkaloids which have a hepatotoxic and carcinogenic effect on humans [77]. Thus, further studies are needed to assess the safety of some species sold in the market. Similar controversies were discussed for the plants sold in a Georgian market where *Symphytum,* also rich in these alkaloids, is sold for consumption [10]. *Arum orientale*, with acrid and irritating properties due to the presence of crystals of oxalic acid, is another controversial species. As described in Appendix 2 only thorough drying and further thermal processing ensures the safe consumption of this plant.

There is a large overlap between medicinal and food species (Appendix 2). This overlap is expressed for example by the use of the same species for teas both for recreational use and medical purposes, and as spices (e.g. *Artemisia, Thymus, Hypericum perforatum*). Medicinal attributes of wild foods are also widely known. Good examples of plant use on a food-medicine continuum include the fruit syrup from *Morus alba* and *M. nigra* or sweets made from the cones of *Pinus kochiana*, which are sweets used for the treatment of coughs and respiratory system diseases. The powder of *Glycyrrhiza glabra* roots and rhizomes added to the traditional Armenian bread (*lavash*) is used for the same ailments. The persistence of such a food-medicine continuum occurs in many societies throughout the world [78, 79], including Eurasia [80–83].

The importance of local products that are often derived from wild food for Armenian economy was already noticed by Pieroni and colleagues [17]. In their paper, they made a list of wild products that could become important trading items to local inhabitants. Some of them, such as products made from the fruits of Rosaceae trees and shrubs and from *Eleagnus* spp., are already on sale in Yerevan. We would go even further and say that the many interesting lacto-fermented Apiaceae made in Yerevan could even become internationally recognised as part of a healthy cuisine, on the aforementioned wave of popularity of lacto-fermented products in general [76]. Pieroni et al. [17] and Slow Food [84] used the term *foodscouting* to describe the activity of looking for valuable local traditional food products. Market surveys play a large role in *foodscouting* as well. In countries with a very rich ethnogastronomic heritage like Armenia, food stalls enable the documentation of new foods and new processing techniques. We advocate for the documentation of plants sold in markets of selected urban centres in all the countries of the world. So far, we lack such documentation from other countries of the Caucasus, Central Asia and many East Asian countries.

Another interesting feature of Caucasian markets is the sale of dried wild vegetables. They are sold either in loose form (e.g. *Ornithogalum hajastanum* in Fig. 3) or entwined into circles for further boiling in winter. Drying wild vegetables and preserving them for winter is a sign of their high cultural importance and has survived as a practise only in few countries, mainly China [85]. In the past it was also recorded in Europe, e.g. in the present territory of Belarus, but the practise is now obsolete [86].

## Conclusions

The Yerevan markets are rich in wild edible and medicinal plants and wild-collected fungi (sold mainly but not only for food). They are similar to other south Asian countries in this respect, and they are richer in edible and medicinal species than European markets. It is particularly worth noting the large number of lacto-fermented products for sale.

Further studies of plants and fungi sold in traditional open markets need to be made in other large towns of the Caucasus as well as in most countries that are not highly industrialised.

## Data Availability

For voucher specimens, see the “Methods” section.

## Appendix 1

The list of the surveyed markets

1. Arabkir Market - farm market, 53 Komitas Ave

2. Open-air market, 49 Marshal Baghramyan Ave

3. Open-air market, Komitas St

4. Parma Supermarket, 79 Marshal Baghramyan Ave

5. Mergelyan Shuka - farm market, 2 Hakob Hakobyan St

6. Shirak Bazar - open-air market, Gyulbenkyan St

7. Yeritsyan & Sons Supermarket, 21 Vahram Papazyan St

8. Gyughamej Eco shop, 18 Hrachya Qochar St

9. Nor Zovq Supermarket, 19 Gulakyan St

10. Aygedzor Supermarket, 2,1 Proshyan St

11. Skyurik Supermarket, 3,1/1 Nalbandyan St

12. Pak Shuka - farm market, 5 Mesrop Mashtots Ave

13. Open-air market, 7 Mesrop Mashtots Ave

14. Open-air market, 31 Mesrop Mashtots Ave

15. Open-air market, 50 Abovyan St

16. Open-air market, 7 Koryun St

17. GUM Market - farm market, 35 Movses Khorenatsi St

18. Open-air market, 52 Arshakunyats Ave

19. Fruit & Vegetable store, 50 Arshakunyats Ave

20. Street shop, 46 Arshakunyats Ave

21. Kayarani Shuka - farm market, Sasuntsi David Square

22. Open-air market and streets shops, Garegin Nzhdeh Square

23. Streets shops, 3 Yeghbayrutian St

24. Open-air market and streets shops, 23-25 Azatutyan Ave

25. Zeytun Market - farm market, 51 Paruyr Sevaki St

26. Fruit & Vegetable store, 123 Armenak Armenakyan St

27. Streets shops, Avan Alma Ata St

28. Streets shops, Marshal Babajanyan st

29. Malatya Agricultural Market, Raffi St

30. Nor Nork Farmers Market, Samvel Safaryan St

31. Palace Farmers Market, Nansen St

32. Streets shops, 14 Mikoyan St

33. Aresh Market, 80 Azatamartikner Ave

34. Streets shops, 111- 113 Muratsan St

35. Farmers Market, 15 Shinararneri St

36. Tsiran Supermarket, 44, 1 Tigran Petrosyan St

37. Gavar Fruit & Vegetable store, 10 Tigran Petrosyan St

## Appendix 2

**Table 3:** Wild plants sold in Yerevan’s markets

Family/species	Main local name	Voucher no. in ERCB and conservation status	Used parts	Ways of use
Acoraceae				
*Acorus calamus* L.	Khnkegheg, baghshtak	Armenian Red List	Rhizomes	MED: Tincture for digestive disorders, respiratory system diseases, inflammatory skin diseases. Decoction against anaemia, diseases of the nervous system, as a lotion against hair loss. Теа for appetite.
Alliaceae				
*Allium atroviolaceum* Boiss.	Karmrasokhuk	13552	Aerial parts, bulb	CUL: Salads, lacto-fermented, spice for dishes and cheese. MED: Fresh leaves for gum pain from the growth of baby teeth, as a multivitamin, fresh bulbs with honey or sugar for respiratory system diseases, baked bulbs for cough.
*Allium rotundum* L.	Dashtaskhtor	13524	Aerial parts, bulb	CUL: Salads, lacto-fermented, spice for dishes and cheese. MED: Fresh leaves for gum pain from the growth of baby teeth, as a multivitamin.
*Allium victorialis* L.	Ghandzil	13484	Aerial parts, bulb	CUL: Salads, lacto-fermented, spice for dishes and cheese. MED: Fresh leaves for gum pain from the growth of baby teeth, as a multivitamin.
Amaranthaceae				
*Amaranthus retroflexus* L.	Havakatar		Young leaves and stems	CUL: Fried. MED: Infusion for diarrhoea, boiled herb for constipation.
*Atriplex prostrata* subsp. *calotheca* (Rafn) M.A.Gust. [syn. *Atriplex hastata* L.]	Mokhrateluk, tal	13556	Young leaves and stems	CUL: Salads, lacto-fermented, fried, filling for pies.
*Atriplex sagittata* Borkh. [syn. *Atriplex nitens* Schkuhr]	Mokhrateluk, tal	13559	Young leaves and stems	CUL: Salads, lacto-fermented, fried, filling for pies.
	Teluk	13509	Young leaves and stems	CUL: Fried. MED: Fresh juice used for stomach and intestine diseases, for the treatment of headaches and constipation.
Apiaceae				
*Angelica tatianae* Bordz.	Bokhni, kekh		Leafstalk, leaves and stems	CUL: Salads, lacto-fermented. MED: Infusion for digestive disorders and respiratory system diseases.
*Anthriscus nemorosa* (Bieb.) Spreng.	Khrkhnduk, trtruk	13523	Young stems, leaves	CUL: Lacto-fermented. MED: Infusion of leaves for digestive disorders and the treatment of skin diseases.
*Astrodaucus orientalis* (L.) Drude	Mandak, astghagazar	13430	Young leaves and stems	CUL: Lacto-fermented, fried. MED: Infusion used in digestive disorders.
*Carum carvi* L.	Qimon, zire, hayots chaman		Seeds	CUL: Spice for dishes, pickles and sujukh (spicy sausage). MED: Infusion for digestive disorders.
*Chaerophyllum aureum L.*	Shushanbanjar, ghmi, mandak	13490	Young leaves and stems, roots	CUL: Aerial parts lacto-fermented, fried. MED: Infusion of roots for digestive disorders.
*Chaerophyllum bulbosum* L.	Shushanbanjar, mandak	13461	Young leaves and stems, bulbous roots	CUL: Lacto-fermented, fried. MED: Infusion of bulbous roots for digestive disorders.
*Daucus carota* L.	Gjazruk	13473	Young leaves and stems, seeds	CUL: Fried. MED: Infusion of seeds for digestive disorders.
*Falcaria vulgaris* Bernh.	Sibekh	13476	Young leaves and stems, roots	CUL: Lacto-fermented, fried. MED: Infusion of leaves to stop bleeding caused by tuberculosis, boiled root with honey and wine for strengthening.
*Ferula szowitziana* DC.	Nardes, bogh	Armenian Red List	Leafstalk, gum	CUL: Lacto-fermented, fried. MED: Infusion against neuritis, epilepsy, kidney stone disease, gum used for digestive disorders and respiratory system diseases.
*Ferulago setifolia* K.Koch	Nardesuk, pirvaz	13564	Young leaves, stems, flowers	CUL: Lacto-fermented, fried, soups.
*Foeniculum vulgare* Mill.	Horom samit	13549	Young stems, seeds	CUL: Fried, used as spice for dishes, pickles, liqueur, candy, sauce. MED: Infusion used in digestive disorders, as a choleretic, carminative and spasmolytic agent.
*Heracleum antasiaticum* Manden	Bldrghan, qegh	13566	Young stems, leafstalk, boots	CUL: Lacto-fermented. MED: Decoction of herb used against liver and gallbladder diseases.
*Heracleum chorodanum* (Hoffm.) DC.	Bldrghan		Young stems, leafstalk	CUL: Lacto-fermented
*Heracleum trachyloma* Fisch. and C.A.Mey.	Bldrghan	13550	Young stems, leafstalk	CUL: Lacto-fermented
*Bilacunaria microcarpa* (M.Bieb.) Pimenov and V.N.Tikhom. [syn. *Hippomarathrum microcarpum* Petrov]	Bokhi, pekhi	13562	Young stems, leafstalk, boots	CUL: Lacto-fermented, fresh juice used for stomach diseases, as hypoglycaemic agent.
*Pimpinella saxifraga* L.	Anison, qoshkhot	13551	Seeds	CUL: Spice for pilaf. MED: Tincture for digestive disorders, respiratory system diseases.
*Prangos ferulacea* (L.) Lindl.	Poli, geli bokhi	13557	Young leaves	CUL: Lacto-fermented, fried.
Araceae				
*Arum orientale* M.Bieb.	Nvik, spitak banjar	13489	Dried and then boiled leaves and rhizomes	CUL: Soups and sauce from dried and then boiled leaves, flour from dried and boiled rhizomes. MED: Powder from dried rhizomes against inflammatory skin diseases, infusion of rhizomes as inflammatory agent for digestive disorders, respiratory system diseases, and for use as an anthelmintic drug.
Asparagaceae				
*Asparagus officinalis* L.	Tsnepak, tsnebek	13553	Young stems	CUL: Salads, fried with eggs. MED: Used as a multivitamin, boiled herb for constipation, infusion as hypoglycaemic and diuretic agent, against inflammatory diseases of the kidneys and bladder.
*Asparagus verticillatus* L.	Tsnepak, tsnebek	13554	Young stems	CUL: Salads, fried with eggs. MED: Used as a multivitamin, boiled herb for constipation, as hypoglycaemic and diuretic agent, against inflammatory diseases of kidneys and bladder.
*Ornithogalum hajastanum* Agapova	Spitak banjar	13469	Dried leaves	CUL: Soup, fried.
*Ornithogalum montanum* Cirillo	Khnjloz	13470	Young leaves and bulbs	CUL: Lacto-fermented, fried.
*Polygonatum glaberrimum* K.Koch	Sindrik	13560	Young leaves and stems, rhizomes	CUL: Salads, lacto-fermented, fried. MED: Fresh rhizomes and leaves used in cosmetology and against skin diseases, boiled leaves and tincture as an antidiabetic remedy.
*Polygonatum multiflorum* (L.) All.	Sindrik		Young leaves and stems, rhizomes	CUL: Salads, lacto-fermented, fried. MED: Fresh rhizomes and leaves used in cosmetology and against skin diseases, boiled leaves and tincture as an antidiabetic remedy.
*Polygonatum orientale* Desf.	Sindrik	13506	Young leaves and stems, rhizomes	CUL: Salads, lacto-fermented, fried. MED: Fresh rhizomes and leaves used in cosmetology and against skin diseases, boiled leaves and tincture as an antidiabetic remedy.
*Puschkinia scilloides* Adams	Alayaz	13532	Fresh and dried leaves	CUL: Friеd, cooked in soup with lentils.
Asphodelaceae				
*Eremurus spectabilis* M.Bieb.	Shresh, shresht	13569	Young leaves, roots	CUL: Salads, fried, lacto-fermented. MED: Infusion of leaves for digestive disorders. Powder from rhizomes used against skin abscesses and cysts.
Asteraceae				
*Achillea millefolium* L.	Hazaraterevuk	13512	Young leaves and stems, flowers, dried herb	CUL: Fresh leaves and fried stems, dried herb as spices for fatty meat, liqueur and tea. MED: Infusion of herbs for digestive disorders and used as appetitive agent against uterine bleeding, as diuretic agent.
*Achillea tenuifolia* Lam.	Hazaraterevuk, chobani banjar		Young leaves and stems, flowers, dried herb	CUL: Fresh leaves and stems fried, dried herb as spices, for liqueur and tea. MED: Infusion of herbs for digestive disorders and as appetitive agent, against uterine bleeding, as diuretic agent.
*Arctium lappa* L.	Kratuk, krotuk	13496	Young leaves, stems, fresh and dried roots	CUL: Young leaves, stems and fresh roots for salads, soup, powder of dried root as coffee. MED: Infusion of roots as antipyretic, diuretic and hypoglycaemic agents, as lotion for hair growth, powder from leaves used against skin abscesses and sores.
*Arctium tomentosum* Mill.	Kratuk	13572	Young leaves, stems, fresh and dried roots	CUL: Young leaves, stems and fresh roots for salads, soup. MED: Infusion of roots as antipyretic, diuretic and hypoglycaemic agents, as lotion for hairs growth, powder from leaves used against skin abscesses and as wound healing agent.
*Artemisia absinthium* L.	Oshindr	13497	Young leaves, herb	CUL: As spices for liqueur and vodka. Dried herbs as mothproofing agent. MED: Infusion of herbs for digestive disorders and as appetitive agent, as anti-inflammatory agent against liver, gallbladder and pancreas diseases, as anthelmintic drug. REP.
*Artemisia austriaca* Jacq.	Oshindr	13414	Young leaves, herb	CUL: As spices for liqueur and vodka. Dried herbs as mothproofing agent. MED: Infusion and tincture of herbs for digestive disorders and as appetitive agent, as anti-inflammatory agent against liver, gallbladder and pancreas diseases, as anthelmintic drug. REP: Against moths.
*Artemisia fragrans* Willd.	Oshindr	13543	Young leaves, herb	CUL: As spices for liqueur and vodka. Dried herbs as mothproofing agent, decorative plant and grass for good luck. MED: Infusion and tincture of herbs for digestive disorders and as appetitive agent, as anti-inflammatory agent for treatment of liver, gallbladder and pancreas diseases as anthelmintic drug. DEC. REP: Against moths. MAGIC.
*Artemisia tournefortiana* Rchb.	Oshindr	13468	Young leaves, herb	CUL: As spices for liqueur and vodka. Dried herbs as mothproofing agent. MED: Infusion and tincture of herbs for digestive disorders and as appetitive agent, as anti-inflammatory agent against liver, gallbladder and pancreas diseases, as anthelmintic drug. REP: Against moths.
*Artemisia vulgaris* L.	Oshindr	13467	Young leaves, herb	As spices for liqueur and vodka. Dried herbs as mothproofing agent. MED: Infusion and tincture of herbs for digestive disorders and as appetitive agent, as anti-inflammatory agent against liver, gallbladder and pancreas diseases, as anthelmintic drug. REP: Against moths.
*Cyanus segetum* Hill. [syn. *Centaurea cyanus* L.]	Terepuk kapuyt	13498	Flowers	CUL: Spice for tea. MED: Decoction for rinsing inflamed eyes. DEC.
*Cichorium intybus* L.	Tchartchatuk, Egherd,	13161	Leaves, stems, roots	CUL: Powder of dried root as coffee or tea. MED: Infusion for digestive disorders and as hypoglycaemic agent, tincture of roots as wound healing agent against snake and scorpion bites.
*Helichrysum rubicundum (*K.Koch) Bornm.	Antaram, anmer tsaghik	13500	Flowers	CUL: Herbal mix for tea. MED: Infusion against liver and gallbladder diseases, as choleretic and appetitive agents. DEC.
*Helichrysum plicatum* DC.	Antaram, anmer tsaghik		Flowers	CUL: Herbal mix for tea. MED: Infusion against liver and gallbladder diseases, as choleretic and appetitive agents. DEC.
*Inula helenium* L.	Heghinei khot, kghmugh		Roots and rhizomes	MED: Decoction as anti-inflammatory, expectorant agent and against respiratory system diseases, as an antipyretic, for digestive disorders and as appetitive agent. Tea with honey against coughs. Decoction or ointment as wound healing agent. DEC.
*Lactuca serriola* L.	Hazar, kathnuk, radika	13477	Young basal leaves	CUL: Salads, in spring as a multivitamin. MED: Infusion against cough, respiratory system diseases, diseases of nervous system. Powder of dried leaves as wound healing agent.
*Leontodon hispidus* L.	Aryutsatam, radika		Young basal leaves	CUL: Salads. MED: Infusion against gallbladder diseases, for teething pain. DEC.
*Leucanthemum vulgare* (Vaill.)Lam.	Spitakatsaghik, eritsuk	13139	Flowers	MED: Infusion and tea mistaken for chamomile. DEC.
*Picris hieracioides* Sibth. and Sm.	Darnitch	13457	Young leaves and stems	CUL: Salads. MED: Boiled leaves for constipation, powder of dried leaves against skin abscesses.
*Podospermum laciniatum* (L.) DC.	Sermnotuk, sindz	13479	Young leaves, roots	CUL: Salads, fried.
*Senecio leucanthemifolius* subsp. *vernalis* (Waldst. and Kit.) Greuter [syn. *Senecio vernalis* Waldst. and Kit.]	Halevoruk	13471	Young leaves	CUL: Fried. MED: Boiled leaves for constipation, as diuretic and choleretic agent.
*Tanacetum polycephalum* subsp. *argyrophyllum (*K.Koch) Podlech [syn. *Tanacetum argyrophyllum* (K.Koch) Tzvel.]	Meghvamushk, lvatsaghik	13501	Flowers	CUL: Spice for pickles and liqueur. Infusion against nephritis.
*Tanacetum vulgare* L.	Tarkavan, мeghvamushk, lvatsaghik	13575	Flowers, herb	CUL: Spice for pickles and liqueur. MED: Herb as anthelmintic agent, against liver, gallbladder, stomach and intestine diseases.
*Taraxacum bessarabicum* (Hornem.) Hand. Mazz.	Khatutik, radika	13576	Young leaves, roots	CUL: Salads, fried. MED: Infusion against digestive disorders, as a diuretic, choleretic, appetitive agent. Fresh leaves against skin diseases and abscesses. Juice of fresh leaves as a multivitamin and against anaemia.
*Taraxacum sonchoides* (D.Don) Sch.Bip. [syn. *Taraxacum montanum* (C.A. Mey.) DC.]	Khatutik, radika		Young leaves, roots	CUL: Salads, fried. MED: Infusion against digestive disorders, as diuretic, choleretic, appetitive agent. Fresh leaves against skin diseases and abscesses. Juice of fresh leaves as a multivitamin and against anaemia.
*Taraxacum officinale* (L.) Weber ex F.H.Wigg.	Khatutik, radika	13475	Young leaves, roots	CUL: Salads, fried. MED: Infusion against digestive disorders, as diuretic, choleretic, appetitive agent. Fresh leaves against skin diseases and abscesses. Juice of fresh leaves as a multivitamin and against anaemia. Latex used against warts.
*Tragopogon coloratus* C.A. Mey.	Sindz, qoshmoruk	13514	Young leaves and stems	CUL: Salads, fried, chewing gum from latex. MED: Latex used to stop bleeding and headaches.
*Tragopogon graminifolius* DC.	Sindz, qoshmoruk		Young leaves and stems	CUL: Salads, fried, chewing gum from latex. MED: Latex used to stop bleeding and headaches.
*Tragopogon dubius* Scop. [syn. *Tragopogon major* Jacq.]	Sindz, qoshmoruk		Young leaves and stems	CUL: Salads, fried, chewing gum from latex.
*Tragopogon pterocarpus* DC.	Sindz, qoshmoruk		Young leaves and stems	CUL: Salads, fried, chewing gum from latex. MED: Use to strengthen immunity, to stop bleeding, against stomach and intestine diseases.
*Tragopogon reticulatus* Boiss. and A. Huet	Sindz, qoshmoruk	13513	Young leaves and stems	CUL: Salads, fried, chewing gum from latex.
*Tragopogon serotinus* Sosn.	Sindz, qoshmoruk		Young leaves and stems	CUL: Salads, fried, chewing gum from latex. MED: Latex used to stop of bleeding, against headaches.
*Tussilago farfara* L.	Tatrak, khochkorik, hazi degh	13502	Leaves	MED: Infusion against respiratory system diseases, cough, as an antipyretic and expectorant agent.
Berberidaceae				
*Berberis vulgaris* L.	Tsoreni, ktsokhur	13485	Fresh and dried fruits, bark of roots and stems, wood	CUL: Jam and liqueur from ripe fruits, as spice for dishes and tea. WOOD: For handicrafts. MED: Infusion of bark against gallbladder, stomach, intestines and eye diseases, against skin abscesses. DEC.
*Berberis orientalis* C.K. Schneid. [this species is now included in *B. vulgaris* according to the Plant List but regarded as separate in Armenian floras]	Tsoreni, ktsokhur	13574	Fresh and dried fruits, wood	CUL: Jam and liqueur from ripe fruits, as spice for dishes and tea. WOOD: For handicrafts. MED: Infusion of bark against gallbladder, stomach, intestine and eye diseases, against skin abscesses. DEC.
Betulaceae				
*Corylus avellana* L.	Tkhleni, tkoghin	13567	Fresh, dried and roasted nuts, leaves, nutshell, wood	CUL: Sweets and candy, nuts. WOOD: For handicrafts. MED: Tea from leaves as diuretic, antipyretic, for boosting the immune system, infusion of leaves against kidney and intestines diseases, as anti-inflammatory agent, ash of nutshell as wound healing agent. Nuts for sexual potency.
Brassicaceae				
*Barbarea vulgaris* R.Br.	Ktsmndzuk	13555	Young and dried leaves, herb	CUL: Salads, fried. MED: Infusion of herb as diuretic, immunity booster, anti-inflammatory agent.
*Capsella bursa-pastoris* (L.) Medik.	Tstapashar	13474	Young leaves	CUL: Salads, fried. MED: Infusion of herbs to prevent bleeding of the uterus, as diuretic, choleretic and wound healing agent. Juice of fresh herbs against kidney, liver and gallbladder diseases.
*Lepidium draba* L.	Khruk, paron banjar	13481	Young leaves	CUL: Fried with eggs.
*Lepidium latifolium* L.	Ghji	13482	Young leaves	CUL: Fried with eggs. MED: Infusion of leaves against skin diseases, nervous disorders and teething pain.
*Rorippa islandica* (Oeder) Borbás	Ktsvich, paron banjar		Young leaves	CUL: Salads.
Cannabaceae				
*Cannabis sativa* L.	Kaneph	13558	Seeds	CUL: In a roasted seed mix of wheat, hemp and flax (aghandz). MED: Powdered seeds with water for boosting the immune system and enhancing sexual potency.
Capparaceae				
*Capparis spinosa* L.	Капар, оtsi dzmeruk	13526	Young flower bud, fruits, roots	CUL: Flower buds used for pickles and as a spice. MED: Infusion of roots used for liver diseases, as hypoglycaemic agent. Compress from pulp of fruits and roots against headaches and joint and muscle pains.
Caprifoliaceae				
*Cephalaria gigantea* (Ledeb.) Bobrov	Ghantapa, jivan	13528	Fresh and dried flowers	CUL: Tea. MED: Infusion as an antipyretic, against colds and coughs, lotion against skin diseases.
*Valeriana officinalis* L.	Katvakhot	13586	Dried roots, rhizomes	MED: Infusion or decoction used for anxiety and stress, for sleep disorders.
*Viburnum opulus* L.	Brnchi	13487	Fresh and dried fruits	CUL: Juice, jam, liqueur, sweets and candy. Fresh and dried fruits used as a multivitamin, diuretic and immune system booster.
Caryophyllaceae				
*Stellaria media* (L.) Cyr.	Tchrtchruk	13478	Young leaves and stems	CUL: Salads, filling for pies (zhingyalov hats).
Colchicaceae				
*Merendera trigyna* Woronow	Khlopuz, dzntsaghik	13507	Blossoming shoots	DEC: Decoration only.
Cornaceae				
*Cornus mas* L.	Hon	13204	Fresh and dried fruits, pips of fruits, wood	CUL: Juice, jam, compote, dry pastille (ttu lavash), liqueur, lacto-fermented. WOOD: For buttons, pips of fruits for bijouterie and chaplets. MED: Jam with tea used to treat digestive disorders, diarrhoea and colds. DEC.
Cucurbitaceae				
*Bryonia alba* L.	Loshtak, arjakhaghogh	13527	Dried roots	MED: Infusion of roots against stomach diseases, haemorrhoids, as immune system booster and enhancer of sexual potency.
Elaeagnaceae				
*Elaeagnus angustifolia* L. [including *Elaeagnus orientalis* L.]	Phshateni	13525	Fresh and dried fruits	CUL: Flour, sweets. MED: Fruits and fruit infusions used to treat digestive disorders and diarrhoea. DEC. Whole branches with fruits for decoration.
*Elaeagnus rhamnoides* (L.) A.Nelson	Chichkhan	13531	Fresh and dried fruits, wood	CUL: Juice, jam, liqueur. MED: Fruits as a multivitamin. Oil of fruits in cosmetology and stomatology for gum disease, as a wound healing agent against skin diseases. DEC. Whole branches with fruits for decoration. WOOD.
Equisetaceae				
*Equisetum arvense* L.	Dziadzet	13517	Herb of green shoot	MED: Infusion of herb as diuretic agent against kidney, bladder, inflammatory and kidney stone diseases.
Ericaceae				
*Vaccinium myrtillus* L.	Hapalaseni	13563	Fresh and dried berries	CUL: Juice, jam, liqueur. MED: Berries as a multivitamin, tea from berries for diarrhoea, eye diseases, and improving eyesight.
Fabaceae				
*Glycyrrhiza glabra* L.	Matutak	13529	Roots, rhizomes	CUL: Sweets. MED: Infusion of roots or powder in bread against colds and coughs, against stomach and intestine diseases.
*Lathyrus pratensis* L.	Tchpruk	13539	Young leaves and stems	CUL: Salad, fried.
*Lathyrus tuberosus* L.	Tchpruk	13542	Tuberous roots	CUL: Boiled.
*Melilotus officinalis* (L.) Pall.	Isharvuyt	13448	Herbs	CUL: Tea. MED: Infusion as a diuretic, against hypertension, diseases of the female reproductive system, fresh juice used for inflammatory diseases of ears and eyes.
*Trifolium pratense* L.	Ereqnuk	13491	Herbs, flowers	CUL: Tea. MED: Infusion as a diuretic, against coughs and diseases of the female reproductive system and the stomach.
*Trifolium repens* L.	Ereqnuk	13499	Flowers	CUL: Tea.
Fagaceae				
*Castanea sativa* Mill.	Shaganak	13530	Nuts, wood	CUL: Fresh, boiled and roasted nuts. Handicrafts. MED: Boiled fruits to strengthen, against lungs and bladder diseases. WOOD.
*Fagus orientalis* Lipsky	Hatchareni	13459	Nuts, wood	CUL: Fresh and roasted nuts. Handicrafts, parquet, door, furniture. WOOD.
Grossulariaceae				
*Ribes uva-crispa* L. [syn. *Grossularia reclinata* (L.) Mill.]	Kokrosheni	13548	Fresh and dried fruits	CUL: Berries for juice, jam, lacto-fermented. MED: Berries as a multivitamin, berry tea against colds and as a diuretic agent.
*Ribes armenum* Pojark.	Hagharjeni	Armenian Red List	Fresh and dried fruits, leaves	CUL: Berries for juice, jam, liqueur, lacto-fermented. Leaves as a tea. MED: Berries as a multivitamin, berry tea against colds, as a diuretic and an antipyretic agent.
*Ribes petraeum* Wulfen [syn. *Ribes biebersteinii* Berland. ex DC.]	Hagharjeni		Fresh and dried fruits, leaves	CUL: Berries for juice, jam, liqueur, lacto-fermented. Leaves as a tea. MED: Berries as a multivitamin, tea of berries against colds, as a diuretic and antipyretic agent.
*Ribes alpinum* L.	Hagharjeni		Fresh and dried fruits, leaves	CUL: Berries for juice, jam, liqueur, lacto-fermented. Leaves as a tea. MED: Berries as a multivitamin, berry tea against colds, as a diuretic and antipyretic agent.
Hypericaceae				
*Hypericum perforatum* L.	Srohund, arevqurik	13511	Dried herbs, oil	CUL: Tea. MED: Infusion against digestive disorders and stomach diseases. Oil against gastric ulcers and skin diseases and for use in cosmetology.
Juglandaceae				
*Juglans regia* L.	Y'nkuzeni	13533	Young, fresh walnut, dried walnuts kernels, dried walnuts partitions, oil, leaves, wood	CUL: Fresh walnuts for jam, dried walnut kernels as sweets, candy, ingredient of savoury dishes and source of edible oil. WOOD: For musical instruments (tar, qyamancha), handicrafts, parquet, door, furniture. REP: Leaves. MED: Tincture of fresh walnuts against hypothyroidism and digestive disorders. Dried walnut kernels with honey as a multivitamin for strengthening immunity, as a sexual potency enhancer and anthelmintic agent. Infusion of dried walnut partitions against headaches, sore throats, diarrhoea, and used as an anthelmintic agent. Oil as an ointment in cosmetology. DEC.
Lamiaceae				
*Leonurus cardiaca* L.	Aryutsagi	13466	Herbs	MED: Tincture and infusion for heart diseases.
*Mentha longifolia* (L.) Huds. Daghd	Daghdz	13494	Fresh and dried leaves, herbs	CUL: Tea, drink, cocktails, as spice for soups, meat dishes, sweets and cheese. MED: Infusion against heartache, headache, digestive disorders and colds.
*Origanum vulgare* L.	Khnkatsaghik, sevakhot	13220	Herbs	CUL: Tea, spice for meat dishes. MED: Infusion against digestive disorders, colds, coughs, and respiratory system diseases. Oil in cosmetology.
*Salvia hydrangea* DC. ex Benth.	Eghespak	13577	Herbs	CUL: Tea.
*Salvia sclarea* L.	Eghespak	13488	Herbs	CUL: Tea. MED: Infusion against teething pain, sore throat and gum disease. Oil in cosmetology.
*Salvia verticillata* L.	Eghespak	13217	Herbs	CUL: Tea. MED: Infusion against teething pain, sore throat and gum disease.
*Stachys palustris* L.	Abeghakhot	13561	Herbs	MED: Infusion or tincture for the treatment of the female reproductive system and irregular periods.
*Teucrium polium* L.	Mariamakhot	13252	Herbs	MED: Infusion for the treatment of the female reproductive system and irregular periods, digestive disorders, stomach and intestines diseases, as eyewash agent.
*Thymus collinus* M.Bieb.	Urts**,** khur	13508	Fresh and dried leaves, herbs	CUL: Tea, as a spice for soups, meat dishes and cheese. MED: Infusion against the digestive disorders, stomach, liver and intestines diseases, colds, respiratory system diseases, hypertension and heartache, as expectorant and antibacterial agent. Oil in cosmetology.
*Thymus kotschyanus* Boiss. and Hohen.	Urts**,** khur	13441	Fresh and dried leaves, herbs	CUL: Tea, as spice for soups, meat dishes and cheese. MED: Infusion against the digestive disorders, stomach, liver and intestine diseases, colds, respiratory system diseases, hypertension and heartache, as expectorant and antibacterial agent. Oil in cosmetology.
*Thymus rariflorus* K.Koch	Urts**,** khur	13503	Fresh and dried leaves, herbs	CUL: Tea, as spice for soups, meat dishes and cheese. MED: Infusion against digestive disorders, stomach, liver and intestine diseases, colds, respiratory system diseases, hypertension and heartache, as expectorant and antibacterial agent. Oil in cosmetology.
*T. transcaucasicus* Ronniger	Urts**,** khur	13545	Fresh and dried leaves, herbs	CUL: Tea, as spice for soups, meat dishes and cheese. MED: Infusion against digestive disorders, stomach, liver and intestine diseases, colds, respiratory system diseases, hypertension and heartache, as expectorant and antibacterial agent. Oil in cosmetology.
*Ziziphora clinopodioides* Lam.	Urtsadaghdz, limoni urts	13546	Fresh and dried leaves, herbs	CUL: Tea, as spice for soups, meat dishes and cheese. MED: Infusion and tea mistaken for thyme.
*Ziziphora clinopodioides* subsp. *rigida* (Boiss.) Rech.f. [syn. *Z. rigida* (Boiss.) Stapf]	Urtsadaghdz, limoni urts	13544	Fresh and dried leaves, herbs	CUL: Tea, as spice for soups, meat dishes and cheese. MED: Infusion and tea mistaken for thyme.
Lythraceae				
*Punica granatum* L.	Nur	13580	Fresh and dried flowers and fruits, dried bark of fruits	CUL: Juice, jam, sweets. Tea from dried flowers and fruits. MED: Infusion of dried bark of fruits against respiratory system diseases and diarrhoea. Dried fruits for handicrafts as a traditional decorative element. DEC.
Malvaceae				
*Malva neglecta* Wallr.	Phiphert	13565	Leaves	CUL: Soups, fried. MED: Infusion against stomach, intestine and kidney diseases, hypertension.
*Malva pusilla* Sm.	Phiphert	13492	Leaves	CUL: Soups, fried. MED: Infusion against stomach, intestine and kidney diseases, hypertension.
*Malva sylvestris* L.	Phiphert	13570	Leaves	CUL: Soups, fried. MED: Infusion against stomach, intestine and kidney diseases, hypertension.
Moraceae				
*Ficus carica* L.	Tzeni	13534	Fresh and dried fruits, latex	CUL: Jam, fresh and dried fruits. MED: Boiled with milk fruits against haemorrhoids. Latex as an anti-wart drug. Fresh and dried fruits for constipation. DEC.
*Morus alba* L.	Tteni spitak	13540	Fresh and dried fruits, leaves, wood	CUL: Juice, jam, Syrup, sweets, vodka. WOOD: For vine barrels, musical instruments (saz). MED: Syrup against coughs. Infusion of leaves for diarrhoea, as hypoglycaemic agent. DEC.
*Morus nigra* L.	Tteni sev		Fresh and dried fruits, leaves, wood	CUL: Juice, jam, Syrup, sweets, vodka. WOOD: For vine barrels, musical instruments (saz). MED: Syrup against coughs. Infusion of leaves as hypoglycaemic agent. More useful than white mulberry. DEC.
Orobanchaceae				
*Phelypaea tournefortii* Desf.	Yot eghbor aryun, chibukh, lala	13379	Flowers	DEC: Decoration only.
Papaveraceae				
*Chelidonium majus* L.	Tsitsernakhot	13472	Herbs, latex	CUL: As a means of protecting plants from aphids. MED: Infusion for neoplasms and as an anti-inflammatory agent against diseases of the female reproductive system. Latex as an anti-wart drug. REP.
Pinaceae				
*Pinus kochiana* Klotzsch ex K.Koch	Sochi	13522	Green female cones, pollen, resin, wood	CUL: Jam and syrup from green female cones as sweets, also MED: Against coughs and diseases of the respiratory system. Powdered pollen against asthma. Resin as an antibacterial agent. WOOD: For handicrafts, doors, saunas.
Plantaginaceae				
*Plantago major* L.	Ezan lezu	13516	Fresh and dried leaves, seeds	CUL: Fried, filling for pies. MED: Infusion or fresh leaves against stomach and intestines diseases. Fresh leaves as a wound healing agent, for the treatment bites of insects. Mucilage of seeds for constipation.
*Plantago lanceolata* L.	Ezan lezu	13515	Fresh leaves, seeds	CUL: Fried, filling for pies. MED: Mucilage of seeds for constipation.
Polygonaceae				
*Rumex acetosa* L.	Trtnjuk	13568	Fresh leaves	CUL: Soups, salads, fried, filling for pies. MED: Infusion against liver diseases, boiled herb for constipation.
*Rumex acetosella* L.	Trtnjuk	13573	Fresh leaves	CUL: Soups, salads, fried, filling for pies. MED: Infusion against liver diseases, boiled herb for constipation.
*Rumex alpinus* L.	Aveluk	13571	Fresh and dried leaves	CUL: Soups, salads, fried, filling for pies. MED: Infusion against stomach and liver diseases and from diarrhoea, boiled herb for constipation. As embrocation against skin diseases and compress against a sore throat.
*Rumex crispus* L.	Aveluk	13240	Fresh and dried leaves	CUL: Soups, salads, fried, filling for pies. MED: Infusion against digestive disorders, stomach and liver diseases and from diarrhoea, cough, boiled herb for constipation. As embrocation against skin and female reproductive system diseases. As compress against a sore throat.
*Rumex tuberosus* L.	Aveluk	13168	Fresh and dried leaves	CUL: Soups, salads, fried, filling for pies. MED: Infusion against digestive disorders, stomach, liver diseases and from diarrhoea, cough, boiled herb for constipation. As embrocation against skin and diseases of the female reproductive system.
Portulaceae				
*Portulaca oleracea* L.	Dandur	13535	Young leaves and stems	CUL: Salads, boiled, lacto-fermented. MED: Infusion against liver, stomach, kidney, and bladder diseases, as hypoglycaemic agent.
Primulaceae				
*Primula veris* subsp. *macrocalyx* (Bunge) Lüdi	Gnarbuk, jangyulum	13187	Flowers	DEC: Decorative plant is a part of a traditional spring game. MED: Infusion of flowers against respiratory system diseases and headache. DEC.
Rhamnaceae				
*Ziziphus jujuba* Mill.	Unab	13480	Fresh and dried fruits	CUL: Fresh and dried fruits. MED: As a multivitamin and diuretic agent. DEC.
Rosaceae				
*Crataegus ambigua* C.A.Mey. ex A.K.Becker [syn. *Crataegus atrosanguinea* Pojark.]	Szni, alotcheni		Fresh and dried fruits, dried flowers	CUL: Juice, jam, liqueur, tea. MED: Tincture and infusion for heart diseases, fruits as multivitamin. DEC.
*Crataegus caucasica* K.Koch	Szni, alotcheni	13579	Fresh and dried fruits, dried flowers	CUL: Juice, jam, liqueur, tea. MED: Tincture and infusion for heart diseases, fruits as multivitamin. DEC.
*Crataegus meyeri* Pojark.	Szni, alotcheni	13582	Fresh and dried fruits, dried flowers	CUL: Juice, jam, liqueur, tea. MED: Tincture and infusion for heart diseases, fruits as multivitamin. DEC.
*Crataegus orientalis* Pall. ex M.Bieb.	Szni, alotcheni	13578	Fresh and dried fruits, dried flowers	CUL: Juice, jam, liqueur, tea. MED: Tincture and infusion for heart diseases, fruits as multivitamin. Мost used. DEC.
*Crataegus pentagyna* Waldst. and Kit. ex Willd.	Szni, alotcheni	13150	Fresh and dried fruits, dried flowers	CUL: Juice, jam, liqueur, tea. MED: Tincture and infusion for heart diseases, fruits as multivitamin. DEC.
*Crataegus rhipidophylla* Gand.	Szni, alotcheni	13581	Fresh and dried fruits, dried flowers	CUL: Juice, jam, liqueur, tea. MED: Tincture and infusion for heart diseases, fruits as multivitamin. DEC.
*Fragaria vesca* L.	Getnamori	13537	Fresh and dried fruits, herbs	CUL: Juice, jam, tea. MED: Infusion or tea of herb against kidney and bladder diseases, as a diuretic agent, fruits as a multivitamin. DEC.
*Malus orientalis* Uglitzk.	Khndzoreni		Fresh and dried fruits	CUL: Juice, jam, lacto-fermented, sweets and candy, vinegar, liqueur. MED: Fruits as a multivitamin and as a source of iron against blood diseases. DEC.
*Mespilus germanica* L.	Zkereni	13505	Fresh fruits	CUL: Lacto-fermented and fresh fruits. MED: Puree of ripe fruits against digestive disorders, constipation and for rebuilding strength after prolonged illness, fruits as a multivitamin. DEC.
*Prunus armeniaca* L.	Tsiraneni	13541	Fresh, frozen and dried fruits, flowers, seeds, wood	CUL: Fruits used to make soup, juice, jam, liqueur, paste, and sweets. Dried flowers and fruits for tea. WOOD: For musical instruments (duduk, tar, qyamancha, zurna) and handicrafts. MED: Fruits as a multivitamin, as a source of potassium against heartache and for constipation. DEC.
*Prunus divaricata* Ledeb.	Saloreni, shloreni		Fresh, frozen and dried fruits, flowers, wood	CUL: Used to make juice, jam, liqueur, lacto-fermented, spice, paste, sweets and candy. WOOD: for musical instruments (saz). MED: Dried fruits against constipation, fruits as a multivitamin. DEC.
*Prunus microcarpa* C.A.Mey.	Baleni		Fresh and dried fruits	CUL: Juice, liqueur, sweets. Fruits as a multivitamin.
*Prunus spinosa* L.	Mamkheni		Fresh, frozen and dried fruits	CUL: Juice, jam, liqueur, lacto-fermented, spice, paste, sweets and candy. MED: Dried fruits against constipation, as a multivitamin, as a diuretic and expectorant agent. DEC.
*Pyrus salicifolia* Pall.	Tandzeni	13536	Fresh and dried fruits	CUL: Juice, jam, liqueur, lacto-fermented, paste, sweets and candy. MED: Fresh and dried fruits used for diarrhoea. DEC.
*Pyrus caucasica* Fed.	Tandzeni	13520	Fresh and dried fruits	CUL: Juice, jam, liqueur, lacto-fermented, paste, sweets and candy. MED: Fresh and dried fruits from diarrhoea. DEC.
*Rosa canina* L.	Masreni	13538	Fresh and dried fruits, petals, oil	CUL: Fresh, dried fruits and petals for juice, jam, liqueur, tea, petals as a spice for sweets and candy. MED: Oil in cosmetology. Infusion of fruits used against colds, stomach and intestine diseases, for strength after prolonged illness, and as a multivitamin and as a diuretic agent. DEC.
*Rosa corymbifera* Borkh.	Masreni	13547	Fresh and dried fruits, petals, oil	CUL: Fresh, dried fruits and petals for juice, jam, liqueur, tea, petals as a spice for sweets and candy. MED: Oil used in cosmetology. Infusion of fruits against colds, stomach and intestine diseases, for strength after prolonged illness, as a multivitamin and as a diuretic agent. DEC.
*Rosa spinosissima* L.	Masreni	13584	Fresh and dried fruits, petals, oil	CUL: Fresh, dried fruits and petals for juice, jam, liqueur, tea, petals as a spice for sweets and candy. MED: Oil in cosmetology. Infusion of fruits against colds, stomach and intestine diseases, for strength after prolonged illness, as a multivitamin and as a diuretic agent. DEC.
*Rubus anatolicus* Focke	Mosheni		Fresh, frozen and dried fruits, leaves	CUL: Fruits for juice, jam, compote, liqueur, sweets, fruits and leaves used as a tea. MED: As a multivitamin, a diuretic agent against colds.
*Rubus caesius* L.	Mosheni	13583	Fresh, frozen and dried fruits, leaves	CUL: Fruits for juice, jam, compote, liqueur, sweets, fruits and leaves used as a tea. MED: As a multivitamin, a diuretic agent against colds.
*Rubus idaeus* L.	Moreni	13585	Fresh, frozen and dried fruits, leaves	CUL: Fruits for juice, jam, compote, liqueur, sweets, fruits and leaves used as a tea. MED: As a multivitamin, a diuretic agent against colds.
*Sorbus aucuparia* L.	Aroseni	13486	Fresh, frozen and dried fruits	CUL: Fruits for compote, liqueur, tea. MED: As a multivitamin, a diuretic and a choleretic agent, infusion for heart muscle treatment. DEC.
Rubiaceae				
*Rubia tinctorum* L.	Toron	13518	Roots, fruits	DEC: Dye for Easter eggs and threads. MED: Infusion of roots used as a diuretic agent against kidney and bladder diseases.
Salicaceae				
*Salix* spp.	Ureni		Branches	WOOD: Withes for baskets, wreaths. Spring garlands on Palm Sunday. DEC.
Tiliaceae				
*Tilia cordata* Mill.	Loreni	13519	Inflorescence, wood	CUL: Tea. WOOD: Handicrafts. MED: Infusion or tea from inflorescence against colds, cough and respiratory system diseases. DEC.
Urticaceae				
*Urtica dioica* L.	Eghinj	13521	Young leaves	CUL: Soups, fried, salads, as a filling for pies. MED: Infusion as a multivitamin, a diuretic agent, for the prevention of bleeding of the uterus, herbs boiled against constipation and haemorrhoids.
Vitaceae				
*Vitis vinifera* L. [syn. *Vitis sylvestris* C.C.Gmel.]	Khaghogh		Young leaves, fresh and dried fruits	CUL: Young leaves for dolma (stuffed leaves with meat), fruits in the form of compote, jam, sweets, candy, wine, syrup, vinegar, raisins. DEC.

*CUL* culinary, *MED* medical, *DEC* decorative, *REP* insect repellent
